# Bis(acetophenone oxime) *O*,*O*′-methyl­ene ether

**DOI:** 10.1107/S160053680803897X

**Published:** 2008-11-26

**Authors:** Yong Zhang, Hong-Jun Zang, Bo-Wen Cheng, Jun Song

**Affiliations:** a School of Materials and Chemical Engineering and Key Laboratory of Hollow Fiber Membrane Materials & Membrane Processes, Tianjin Polytechnic University, Tianjin 300160, People’s Republic of China

## Abstract

In the mol­ecule of the title compound, C_17_H_18_N_2_O_2_, the dihedral angle between the aromatic rings is 74.26 (3)°. The oxime units are oriented at dihedral angles of 7.66 (3) and 33.06 (3)° with respect to the adjacent rings, and they have *E* configurations about the C=N bonds.

## Related literature

For general background on oximes and their varied applications, see: Jones *et al.* (1961 ▶); Schrauzer & Kohnle (1964 ▶); Hashemi *et al.* (2006 ▶); Ghiasvand *et al.* (2004 ▶, 2005 ▶); Kakanejadifard *et al.* (2007 ▶); Otsuka Pharmaceutical Co Ltd (1981 ▶); Chertanova *et al.* (1994 ▶).
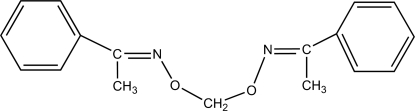


## Experimental

### 

#### Crystal data

C_17_H_18_N_2_O_2_
*M* *_r_* = 282.33
Monoclinic, 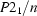

*a* = 9.875 (2) Å
*b* = 8.8409 (18) Å
*c* = 17.290 (4) Å
β = 101.13 (3)°
*V* = 1481.1 (6) Å^3^
*Z* = 4
Mo *K*α radiation
μ = 0.08 mm^−1^
*T* = 113 (2) K
0.14 × 0.04 × 0.04 mm

#### Data collection

Rigaku Saturn diffractometer
Absorption correction: multi-scan (*CrystalClear*; Rigaku/MSC, 2005 ▶) *T*_min_ = 0.988, *T*_max_ = 0.997
9665 measured reflections
2612 independent reflections
1724 reflections with *I* > 2σ(*I*)
*R*_int_ = 0.104

#### Refinement

*R*[*F* ^2^ > 2σ(*F* ^2^)] = 0.045
*wR*(*F* ^2^) = 0.097
*S* = 0.96
2612 reflections
193 parameters
H-atom parameters constrained
Δρ_max_ = 0.23 e Å^−3^
Δρ_min_ = −0.19 e Å^−3^

### 

Data collection: *CrystalClear* (Rigaku/MSC, 2005 ▶); cell refinement: *CrystalClear*; data reduction: *CrystalClear*; program(s) used to solve structure: *SHELXS97* (Sheldrick, 2008 ▶); program(s) used to refine structure: *SHELXL97* (Sheldrick, 2008 ▶); molecular graphics: *SHELXTL* (Sheldrick, 2008 ▶); software used to prepare material for publication: *SHELXTL*.

## Supplementary Material

Crystal structure: contains datablocks I, global. DOI: 10.1107/S160053680803897X/hk2580sup1.cif

Structure factors: contains datablocks I. DOI: 10.1107/S160053680803897X/hk2580Isup2.hkl

Additional supplementary materials:  crystallographic information; 3D view; checkCIF report

## Figures and Tables

**Fig. 1.**
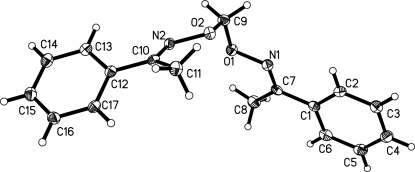
The molecular structure of the title molecule, with the atom-numbering scheme. Displacement ellipsoids are drawn at the 30% probability level.

## supplementary crystallographic information

### Comment

Some oximes are widely used for various purposes in organic, inorganic, bioinorganic, pigment, analytical, dyes and medical chemistry (Jones *et al.*,  1961; Schrauzer & Kohnle, 1964; Hashemi *et al.*,  2006; Ghiasvand *et al.*,  2004; Ghiasvand *et al.*,  2005; Kakanejadifard *et al.*, 2007). Methylene dioximes are important chemicals useful as metal capturers, and antiinflammatory and antibacterial agents (Otsuka Pharmaceutical Co Ltd, 1981). We report herein the synthesis and crystal structure of the title compound.

In the molecule of the title compound (Fig. 1), the bond lengths and angles are within normal ranges. Rings A (C1-C6) and B (C12-C17) are, of course, planar, and they are oriented at a dihedral angle of 74.26 (3)°. The (C1-C7-N1-O1) and (C12/C10/N2/O2) moieties are oriented with respect to the adjacent rings at dihedral angles of 7.66 (3)° and 33.06 (3)°, respectively. The oxime moieties have E configurations [C1-C7-N1-O1 178.38 (12)° and C12-C10-N2-O2 179.02 (10)°; Chertanova *et al.*,  1994].

### Experimental

For the preparation of the title compound, the acetophenone oxime (0.5 mmol) was dissolved in dichloromethane (3.5 ml). [bmim]BF_4_ (0.2269 g, 0.1 mmol) and sodium hydroxide (0.167 g) were added. The reaction mixture was stirred at room temperature for 30 min. The mixture was washed with water (10 ml) and extracted with CH_2_Cl_2_ (15 ml). The combined organic layers were dried over anhydrous sodium sulfate, filtered, and evaporated to dryness *in vacuo*. The product was purified by chromatography on silica (200–300 mesh). Elution with a mixture of petroleum ether and ethyl acetate [1/20(*v*/*v*)] afforded the methylene dioxime. Crystals suitable for X-ray analysis were obtained by slow evaporation of a water solution.

### Refinement

H atoms were positioned geometrically, with C-H = 0.93, 0.97 and 0.96 Å, respectively for aromatic, methylene and methyl H atoms, and constrained to ride on their parent atoms with U_iso_(H) = xU_eq_(C), where x = 1.5 for methyl H, and x = 1.2 for all other H atoms.

### Crystal data

**Table:**

C_17_H_18_N_2_O_2_	*F*_000_ = 600
*M**_r_* = 282.33	*D*_x_ = 1.266 Mg m^−^^3^
Monoclinic, *P*2_1_/*n*	Mo *K*α radiation λ = 0.71073 Å
Hall symbol: -P 2yn	Cell parameters from 2756 reflections
*a* = 9.875 (2) Å	θ = 2.4–27.5º
*b* = 8.8409 (18) Å	µ = 0.08 mm^−^^1^
*c* = 17.290 (4) Å	*T* = 113 (2) K
β = 101.13 (3)º	Prism, colorless
*V* = 1481.1 (6) Å^3^	0.14 × 0.04 × 0.04 mm
*Z* = 4	

### Data collection

**Table:**

Rigaku Saturn diffractometer	2612 independent reflections
Radiation source: rotating anode	1724 reflections with *I* > 2σ(*I*)
Monochromator: confocal	*R*_int_ = 0.105
*T* = 113(2) K	θ_max_ = 25.0º
ω scans	θ_min_ = 2.2º
Absorption correction: multi-scan(CrystalClear; Rigaku/MSC, 2005)	*h* = −11→11
*T*_min_ = 0.988, *T*_max_ = 0.997	*k* = −9→10
9665 measured reflections	*l* = −20→16

### Refinement

**Table:**

Refinement on *F*^2^	Hydrogen site location: inferred from neighbouring sites
Least-squares matrix: full	H-atom parameters constrained
*R*[*F*^2^ > 2σ(*F*^2^)] = 0.045	*w* = 1/[σ^2^(*F*_o_^2^) + (0.0345*P*)^2^] where *P* = (*F*_o_^2^ + 2*F*_c_^2^)/3
*wR*(*F*^2^) = 0.097	(Δ/σ)_max_ = 0.001
*S* = 0.96	Δρ_max_ = 0.23 e Å^−^^3^
2612 reflections	Δρ_min_ = −0.19 e Å^−^^3^
193 parameters	Extinction correction: SHELXL97 (Sheldrick, 2008), Fc^*^=kFc[1+0.001xFc^2^λ^3^/sin(2θ)]^-1/4^
Primary atom site location: structure-invariant direct methods	Extinction coefficient: 0.016 (2)
Secondary atom site location: difference Fourier map	

### Special details

**Table:**

Geometry. All e.s.d.'s (except the e.s.d. in the dihedral angle between two l.s. planes) are estimated using the full covariance matrix. The cell e.s.d.'s are taken into account individually in the estimation of e.s.d.'s in distances, angles and torsion angles; correlations between e.s.d.'s in cell parameters are only used when they are defined by crystal symmetry. An approximate (isotropic) treatment of cell e.s.d.'s is used for estimating e.s.d.'s involving l.s. planes.
Refinement. Refinement of *F*^2^ against ALL reflections. The weighted *R*-factor *wR* and goodness of fit *S* are based on *F*^2^, conventional *R*-factors *R* are based on *F*, with *F* set to zero for negative *F*^2^. The threshold expression of *F*^2^ > σ(*F*^2^) is used only for calculating *R*-factors(gt) *etc*. and is not relevant to the choice of reflections for refinement. *R*-factors based on *F*^2^ are statistically about twice as large as those based on *F*, and *R*- factors based on ALL data will be even larger.

### Fractional atomic coordinates and isotropic or equivalent isotropic displacement parameters (Å^2^)

**Table:**

	*x*	*y*	*z*	*U*_iso_*/*U*_eq_	
O1	0.36208 (10)	0.65077 (13)	0.02442 (6)	0.0284 (3)	
O2	0.14145 (11)	0.61984 (13)	0.05282 (6)	0.0284 (4)	
N1	0.39081 (13)	0.79925 (17)	0.05526 (7)	0.0270 (4)	
N2	0.15155 (13)	0.46386 (16)	0.07470 (8)	0.0257 (4)	
C1	0.55638 (16)	0.9740 (2)	0.11238 (8)	0.0247 (4)	
C2	0.45724 (17)	1.0888 (2)	0.10304 (9)	0.0274 (5)	
H2	0.3675	1.0676	0.0774	0.033*	
C3	0.48996 (17)	1.2324 (2)	0.13110 (9)	0.0315 (5)	
H3	0.4223	1.3070	0.1246	0.038*	
C4	0.62333 (18)	1.2665 (2)	0.16904 (9)	0.0357 (5)	
H4	0.6457	1.3636	0.1879	0.043*	
C5	0.72255 (18)	1.1545 (2)	0.17853 (10)	0.0360 (5)	
H5	0.8121	1.1765	0.2041	0.043*	
C6	0.69013 (17)	1.0102 (2)	0.15045 (9)	0.0328 (5)	
H6	0.7583	0.9362	0.1570	0.039*	
C7	0.52021 (16)	0.8199 (2)	0.08160 (9)	0.0251 (4)	
C8	0.62975 (17)	0.7028 (2)	0.08213 (10)	0.0362 (5)	
H8A	0.5891	0.6128	0.0565	0.054*	
H8B	0.6721	0.6797	0.1356	0.054*	
H8C	0.6983	0.7408	0.0546	0.054*	
C9	0.21947 (16)	0.6427 (2)	−0.00642 (9)	0.0283 (5)	
H9A	0.2020	0.5603	−0.0441	0.034*	
H9B	0.1899	0.7358	−0.0343	0.034*	
C10	0.09103 (15)	0.4381 (2)	0.13271 (9)	0.0231 (4)	
C11	0.02188 (17)	0.5564 (2)	0.17390 (10)	0.0329 (5)	
H11A	0.0883	0.6006	0.2157	0.049*	
H11B	−0.0509	0.5107	0.1954	0.049*	
H11C	−0.0159	0.6337	0.1369	0.049*	
C12	0.09387 (15)	0.2791 (2)	0.15882 (9)	0.0241 (4)	
C13	0.09137 (16)	0.1598 (2)	0.10568 (9)	0.0278 (5)	
H13	0.0876	0.1804	0.0526	0.033*	
C14	0.09444 (16)	0.0116 (2)	0.13108 (10)	0.0324 (5)	
H14	0.0931	−0.0670	0.0952	0.039*	
C15	0.09958 (16)	−0.0200 (2)	0.20996 (10)	0.0327 (5)	
H15	0.1010	−0.1199	0.2269	0.039*	
C16	0.10255 (16)	0.0964 (2)	0.26369 (10)	0.0303 (5)	
H16	0.1065	0.0751	0.3167	0.036*	
C17	0.09954 (15)	0.2444 (2)	0.23818 (9)	0.0265 (5)	
H17	0.1013	0.3224	0.2744	0.032*	

### Atomic displacement parameters (Å^2^)

**Table:**

	*U*^11^	*U*^22^	*U*^33^	*U*^12^	*U*^13^	*U*^23^
O1	0.0275 (7)	0.0271 (9)	0.0310 (7)	0.0022 (6)	0.0069 (5)	−0.0017 (5)
O2	0.0292 (7)	0.0262 (9)	0.0310 (7)	0.0010 (6)	0.0085 (5)	0.0017 (5)
N1	0.0287 (8)	0.0268 (10)	0.0262 (8)	0.0008 (7)	0.0071 (6)	0.0000 (6)
N2	0.0248 (8)	0.0211 (10)	0.0305 (8)	−0.0004 (7)	0.0031 (6)	0.0003 (7)
C1	0.0231 (9)	0.0314 (13)	0.0209 (9)	0.0014 (9)	0.0073 (7)	0.0047 (8)
C2	0.0244 (9)	0.0333 (13)	0.0246 (9)	−0.0004 (9)	0.0050 (7)	−0.0009 (8)
C3	0.0325 (10)	0.0335 (14)	0.0296 (10)	0.0025 (9)	0.0085 (8)	0.0005 (8)
C4	0.0412 (12)	0.0374 (14)	0.0286 (10)	−0.0098 (10)	0.0073 (8)	0.0012 (9)
C5	0.0265 (10)	0.0471 (16)	0.0328 (11)	−0.0084 (10)	0.0018 (8)	0.0047 (9)
C6	0.0252 (10)	0.0420 (15)	0.0317 (10)	0.0013 (9)	0.0070 (7)	0.0069 (9)
C7	0.0250 (9)	0.0308 (12)	0.0205 (9)	0.0053 (8)	0.0068 (7)	0.0052 (8)
C8	0.0293 (10)	0.0370 (14)	0.0416 (11)	0.0072 (9)	0.0051 (8)	0.0005 (9)
C9	0.0278 (10)	0.0332 (13)	0.0236 (10)	−0.0026 (8)	0.0044 (8)	0.0017 (8)
C10	0.0173 (9)	0.0276 (12)	0.0237 (9)	−0.0008 (8)	0.0024 (7)	−0.0040 (8)
C11	0.0334 (10)	0.0299 (13)	0.0369 (10)	0.0031 (9)	0.0100 (8)	−0.0027 (8)
C12	0.0167 (9)	0.0258 (12)	0.0294 (10)	−0.0002 (8)	0.0038 (7)	−0.0014 (8)
C13	0.0255 (10)	0.0300 (14)	0.0288 (10)	−0.0012 (9)	0.0075 (7)	−0.0028 (8)
C14	0.0301 (10)	0.0265 (13)	0.0411 (11)	0.0014 (9)	0.0082 (8)	−0.0058 (9)
C15	0.0252 (10)	0.0278 (13)	0.0440 (11)	0.0011 (9)	0.0037 (8)	0.0050 (9)
C16	0.0254 (10)	0.0332 (14)	0.0310 (10)	−0.0004 (9)	0.0023 (8)	0.0044 (9)
C17	0.0203 (9)	0.0278 (13)	0.0309 (10)	−0.0008 (8)	0.0041 (7)	−0.0059 (8)

### Geometric parameters (Å, °)

**Table:**

O1—C9	1.4081 (17)	C8—H8B	0.9600
O1—N1	1.4246 (17)	C8—H8C	0.9600
O2—C9	1.4100 (19)	C9—H9A	0.9700
O2—N2	1.4283 (17)	C9—H9B	0.9700
N1—C7	1.2842 (19)	C10—C12	1.476 (2)
N2—C10	1.283 (2)	C10—C11	1.502 (2)
C1—C6	1.394 (2)	C11—H11A	0.9600
C1—C2	1.397 (2)	C11—H11B	0.9600
C1—C7	1.481 (2)	C11—H11C	0.9600
C2—C3	1.375 (2)	C12—C13	1.396 (2)
C2—H2	0.9300	C12—C17	1.397 (2)
C3—C4	1.386 (2)	C13—C14	1.381 (2)
C3—H3	0.9300	C13—H13	0.9300
C4—C5	1.380 (2)	C14—C15	1.383 (2)
C4—H4	0.9300	C14—H14	0.9300
C5—C6	1.381 (2)	C15—C16	1.383 (2)
C5—H5	0.9300	C15—H15	0.9300
C6—H6	0.9300	C16—C17	1.379 (2)
C7—C8	1.496 (2)	C16—H16	0.9300
C8—H8A	0.9600	C17—H17	0.9300
			
C9—O1—N1	107.51 (12)	O1—C9—H9A	109.1
C9—O2—N2	108.10 (12)	O2—C9—H9A	109.1
C7—N1—O1	112.12 (13)	O1—C9—H9B	109.1
C10—N2—O2	111.03 (14)	O2—C9—H9B	109.1
C6—C1—C2	117.83 (17)	H9A—C9—H9B	107.9
C6—C1—C7	121.41 (15)	N2—C10—C12	115.07 (16)
C2—C1—C7	120.75 (14)	N2—C10—C11	124.78 (17)
C3—C2—C1	121.18 (15)	C12—C10—C11	120.15 (16)
C3—C2—H2	119.4	C10—C11—H11A	109.5
C1—C2—H2	119.4	C10—C11—H11B	109.5
C2—C3—C4	120.31 (17)	H11A—C11—H11B	109.5
C2—C3—H3	119.8	C10—C11—H11C	109.5
C4—C3—H3	119.8	H11A—C11—H11C	109.5
C5—C4—C3	119.23 (18)	H11B—C11—H11C	109.5
C5—C4—H4	120.4	C13—C12—C17	118.25 (17)
C3—C4—H4	120.4	C13—C12—C10	121.45 (16)
C4—C5—C6	120.65 (16)	C17—C12—C10	120.30 (15)
C4—C5—H5	119.7	C14—C13—C12	120.74 (16)
C6—C5—H5	119.7	C14—C13—H13	119.6
C5—C6—C1	120.80 (17)	C12—C13—H13	119.6
C5—C6—H6	119.6	C13—C14—C15	119.97 (17)
C1—C6—H6	119.6	C13—C14—H14	120.0
N1—C7—C1	114.38 (15)	C15—C14—H14	120.0
N1—C7—C8	124.93 (16)	C16—C15—C14	120.26 (18)
C1—C7—C8	120.69 (14)	C16—C15—H15	119.9
C7—C8—H8A	109.5	C14—C15—H15	119.9
C7—C8—H8B	109.5	C17—C16—C15	119.67 (17)
H8A—C8—H8B	109.5	C17—C16—H16	120.2
C7—C8—H8C	109.5	C15—C16—H16	120.2
H8A—C8—H8C	109.5	C16—C17—C12	121.11 (16)
H8B—C8—H8C	109.5	C16—C17—H17	119.4
O1—C9—O2	112.27 (12)	C12—C17—H17	119.4
			
C9—O1—N1—C7	−176.97 (13)	N1—O1—C9—O2	−79.77 (15)
C9—O2—N2—C10	175.14 (11)	N2—O2—C9—O1	−78.02 (14)
C6—C1—C2—C3	−0.6 (2)	O2—N2—C10—C12	179.02 (10)
C7—C1—C2—C3	−179.71 (15)	O2—N2—C10—C11	−1.56 (19)
C1—C2—C3—C4	0.4 (3)	N2—C10—C12—C13	−32.7 (2)
C2—C3—C4—C5	−0.2 (3)	C11—C10—C12—C13	147.83 (15)
C3—C4—C5—C6	0.2 (3)	N2—C10—C12—C17	147.12 (15)
C4—C5—C6—C1	−0.4 (3)	C11—C10—C12—C17	−32.3 (2)
C2—C1—C6—C5	0.6 (2)	C17—C12—C13—C14	0.0 (2)
C7—C1—C6—C5	179.70 (16)	C10—C12—C13—C14	179.83 (14)
O1—N1—C7—C1	178.38 (12)	C12—C13—C14—C15	0.2 (2)
O1—N1—C7—C8	−1.2 (2)	C13—C14—C15—C16	−0.4 (2)
C6—C1—C7—N1	173.26 (15)	C14—C15—C16—C17	0.4 (2)
C2—C1—C7—N1	−7.7 (2)	C15—C16—C17—C12	−0.2 (2)
C6—C1—C7—C8	−7.1 (2)	C13—C12—C17—C16	0.0 (2)
C2—C1—C7—C8	171.95 (15)	C10—C12—C17—C16	−179.87 (14)
